# No role for estrogen receptor 1 gene intron 1 Pvu II and exon 4 C325G polymorphisms in migraine susceptibility

**DOI:** 10.1186/1471-2350-7-12

**Published:** 2006-02-28

**Authors:** Natalie J Colson, Rod A Lea, Sharon Quinlan, Lyn R Griffiths

**Affiliations:** 1 Genomics Research Centre, School of Health Science, Griffith University Gold Coast, PMB 50, Gold Coast Mail Centre, Queensland, 9726, Australia; 2 Institute of Environmental Science and Research, 34 Kenepuru Drive, Porirua Wellington, New Zealand

## Abstract

**Background:**

We have previously reported an association between the estrogen receptor 1 (ESR1) gene exon 8 G594A polymorphism and migraine susceptibility in two independent Australian cohorts. In this paper we report results of analysis of two further single nucleotide polymorphisms (SNPs) in the ESR1 gene in the same study group, the T/C *Pvu *II SNP in intron 1 and the C325G SNP in exon 4, as well as results of linkage disequilibrium (LD) analysis on these markers.

**Methods:**

We investigated these variants by case-control association analysis in a cohort of 240 migraineurs and 240 matched controls. The SNPs were genotyped using specific restriction enzyme assays. Results were analysed using contingency table methods incorporating the chi-squared statistic. LD results are presented as D' statistics with associated *P *values.

**Results:**

We found no evidence for association of the *Pvu *II T/C polymorphism and the C325G polymorphism and migraine susceptibility and no evidence for LD between these two SNPs and the previously implicated exon 8 G594A marker.

**Conclusion:**

We have found no role for the polymorphisms in intron 1 and exon 4 with migraine susceptibility. To further investigate our previously implicated exon 8 marker, we suggest the need for studies with a high density of polymorphisms be undertaken, with particular focus on markers in LD with the exon 8 marker.

## Background

Migraine is a frequent debilitating neurological disorder that affects a significant proportion of the population. The pathophysiology of migraine is not fully understood, although cortical hypersensitivity, vasospasm, neurotransmitters, especially serotonin (5-hydroxytryptamine, 5-HT), platelet activation and sympathetic hyperactivity all appear to play a part, whether as part of the primary triggering event, or as a response mechanism. In the absence of any biological marker, migraine diagnosis is currently based on subjective criteria alone. To further compound the problem, treatment efficacy is limited. Migraine imparts a significant burden on society, both socially and financially. The World Health Organization has identified migraine among the world's top 20 leading causes of disability, with an impact that extends far beyond individual suffering [1].

There is significant evidence from family and twin studies to indicate a strong genetic component to migraine. The current understanding of migraine is that it is a polygenic multifactorial disorder [2]. It has been postulated that genetic factors set the individual migraine threshold, with environmental influences playing a modulating role [3]. It is likely that many genes may provide an important although small contribution to an individual's migraine susceptibility [4]. The identification of migraine susceptibility genes has been the focus of substantial research to date and could eventually lead to improved treatments and greater understanding of the disorder. Several loci have shown promise, although these need to be followed up by both replication and functional studies to determine a definitive causative role [5-15].

The estrogen receptor 1 (ESR1) gene is a potential migraine candidate due to the well-known hormonal influence on migraine susceptibility. Migraines in women frequently occur during the childbearing years and are often influenced by significant hormonal milestones. The fluctuating hormone levels of the menstrual cycle have been implicated in migraine but a definitive role is yet to be established [16]. It has been suggested that factors additional to the circulating hormone levels may be at play [17]. Thus, we considered that variation in the ESR1 gene may confer increased migraine risk. To investigate the potential role of ESR1 in migraine we conducted an association study of the ESR1 G594A polymorphism (rs2228480) and migraine in two independent case-control groups. These previously reported results showed that individuals who carried the 594A allele were twice as likely to suffer from migraine than those who carried the 594G allele [18]. The G594A polymorphism is in exon 8 of ESR1, a gene of approximately 295 kilobases in size and consisting of 8 exons. It is a synonymous polymorphism with no associated amino acid change, consequently it is unlikely that this polymorphism is causative, but may be in linkage disequilibrium (LD) with an unknown causative variant. In this study we have analysed two further single nucleotide polymorphisms (SNPs) in ESR1 in the same study group, the *Pvu *II C/T SNP in intron 1 (rs2234693,) and the C325G SNP in exon 4 (rs1801132) which is located in the hormone binding region. The *Pvu *II locus has been associated with variation in estradiol levels in post menopausal women [19] and with an increased risk of stroke in men [20]. Interestingly both estrogen withdrawal and high estrogen concentrations have been implicated in migraine susceptibility in women [16], and there is evidence for an increased risk of stroke in MA sufferers [21]. It has been reported that the C325G SNP may play a role in calcium metabolism [22] and susceptibility to breast cancer [23,24] a disease in which hormones play a role. The minor allele at each of these SNPs has been shown to have a frequency of >20% as determined in Australian and other Caucasian populations [25,26].

## Methods

### Subjects

Research was approved by the Griffith University Ethics Committee for experimentation on human subjects. Informed consent was obtained from all participants prior to commencement. All were of Caucasian origin, and were recruited from the east coast of Australia through the Genomics Research Centre's patient clinic whereby each participant was interviewed, and completed a detailed questionnaire on personal and family medical history, migraine symptoms, age of onset, frequency, severity, treatment and response, and migraine triggers as previously described [27,28]. Migraine was diagnosed by a clinical neurologist as either migraine with aura (MA), or migraine without aura (MO) based strictly on the widely accepted criteria specified by the International Headache Society [29]. The study population was comprised of 240 migraineurs and 240 unrelated control individuals. To minimize potential bias from population stratification, the control group was matched for sex, age (+/- 5 years), and ethnicity.

### Genotyping

**Figure 1:**
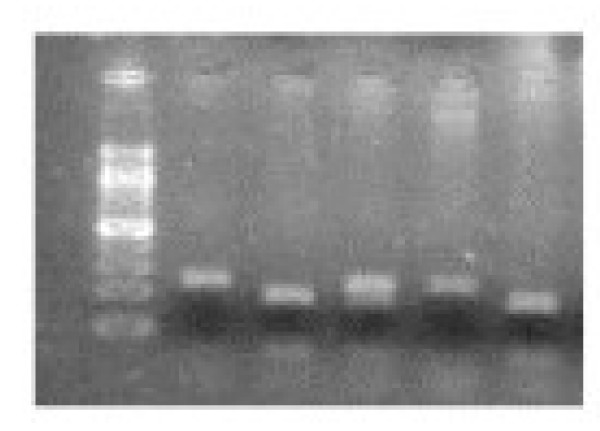
Electrophoretogram of ESR1 *Pvu *II genotypes. Lane 1 shows the 100 bp ladder. Lanes 2 & 5 show 239 bp fragments representing TT genotypes. Lanes 3 & 6 show 99 and faint 39 bp fragments representing CC genotypes. Lane 4 shows 239, 99 and faint 39 bp fragments representing TC genotypes.

Both markers were amplified using polymerase chain reaction (PCR). For the ESR1 *Pvu *II marker primers used were those previously described by Lai et al., 2002 resulting in a 239 bp fragment following PCR [30]. The 20 μl PCR reaction mix contained 40 ng genomic DNA, 0.2 μM of each primer, 1 × PCR buffer, 2 mM MgCl_2_, 0.2 mM dNTPs and 0.2 μl *Taq *polymerase (5 U/μl). Thermocycler conditions were 94°C for 2 minutes, 35 cycles of 94°C for 30 seconds, 55°C for 1 minute, with a final step of 72°C for 5 minutes. The C allele introduces a restriction site for the *Pvu *II enzyme, resulting in fragments of 140 and 99 base pairs. Following amplification, 10 μl of product was digested with *Pvu *II overnight at 37°C. After digestion, the product was loaded into a 2% high-resolution agarose gel stained with ethidium bromide and electrophoresed at 90 V for 30 minutes. An undigested sample indicated presence of the T allele. An electrophoretogram of the digested PCR product illustrating all genotypes appears in Figure 1.

**Figure 2:**
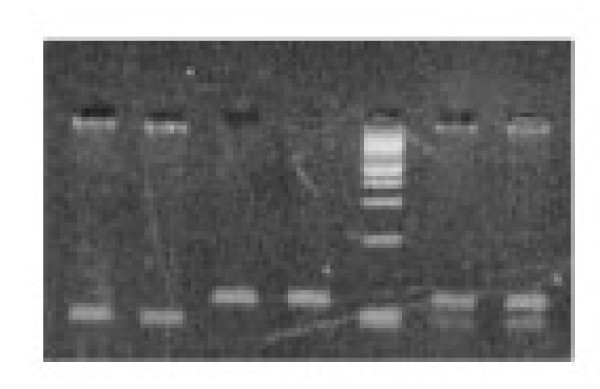
Electrophoretogram of ESR4 C325G genotypes. Lanes 1 & 2 show 99 bp fragments representing GG genotypes (NB. the 21 bp fragment is not visible). Lanes 3 & 4 show 120 bp fragments representing the CC genotypes. Lane 5 shows the 100 bp ladder. Lanes 6 & 7 show the 120 bp & 99 bp fragments representing GC heterozygotes.

The ESR1 C325G marker is a *Hinf*I restriction fragment length polymorphism (RFLP) [25]. Primers used were those previously described with minor modifications [31], (F 5'AGC CCG CTC ATG ATC AAA CG 3' R 5' GGA TCA TAC TCG GAA TAG AGA AT 3') resulting in a 120 base pair fragment following PCR. The 20 μl PCR reaction mix contained 50 ng genomic DNA, 0.3 μM of each primer, 1 × PCR buffer, 2.25 mM MgCl_2_, 0.2 mM dNTPs and 0.2 μl *Taq *polymerase (5 U/μl). Thermocycler conditions were 94°C for 5 minutes, 30 cycles of 94°C for 30 seconds, 62°C for 1 minute and 72°C, with a final step of 72°C for 5 minutes. The G allele at codon 325 in the ESR1 gene introduces a restriction site for the *Hinf*I enzyme, resulting in fragments of 99 and 21 base pairs. Following amplification, 10 μl of product was digested with *Hinf*I overnight at 37°C. After digestion, the product was loaded into a 5% ultra high-resolution agarose gel stained with ethidium bromide and electrophoresed at 90 V for 60 minutes. An undigested sample indicated presence of the 594C allele. An electrophoretogram of the digested PCR product illustrating all genotypes appears in Figure 2. To reduce the likelihood of genotyping error, random repeat samples and negative controls were included in both assays.

### Statistical analysis

Genotype data and allele frequencies were compared between the migraine case and control groups using standard chi-square analysis. Due to multiple testing, the Bonferroni correction for 5 tests was applied, which set the level of significance at 0.01 (ie. 0.05/5).

Linkage disequilibrium between the ESR1 intron 1, the ESR1 exon 4 polymorphism and the previously reported exon 8 polymorphism that was tested in the same study group was analysed using the 2LD program [13]. LD results are presented as D' and P values.

## Results and Discussion

### Case Control analysis

Statistical analysis of the *Pvu *II marker revealed no significant difference between genotyped migraineurs and the matched control group with regard to genotype frequencies (χ^2 ^= 1.94, *P *= 0.38) and allele frequencies (χ^2 ^= 1.02, *P *= 0.31). Furthermore, no significant difference was seen when the migraine population was subdivided into MA (genotype frequencies χ^2 ^= 3.53, *P *= 0.17, allele frequencies χ^2 ^= 0.32 0, *P *= 0.57) and MO (genotype frequencies χ^2 ^= 1.66, *P *= 0.44, allele frequencies χ^2 ^= 1.52, *P *= 0.22), although the increased frequency of the CC genotype in MO (30%) compared to MA (20%) may warrant follow-up in a larger study group. There was no statistically significant difference in the migraine and control groups with regard to males (genotype frequencies χ^2 ^= 2.36, *P *= 0.31, allele frequencies χ^2 ^= 2.36, *P *= 0.12) and females (genotype frequencies χ^2 ^= 0.65, *P *= 0.72, allele frequencies χ^2 ^= 0.03, *P *= 0.86). With regard to male frequencies, it was interesting to note that there was a higher frequency of the CC genotype in male migraineurs (27%) compared to the male control group (19%). While this analysis did not reach statistical significance due to small numbers in the male subgroup, it may warrant further investigation in a larger study group, particularly in view of the previously reported role of the CC genotype in increased stroke risk in males [20] and the potential relationship between migraine and stroke [21,32-35]. Allele frequencies did not deviate from Hardy Weinberg Equilibrium in both case and control groups (at *P *= 0.4, *P *= 0.6) and were similar to previously reported frequencies [26]. Frequency distribution appears in Table 1.

**Table 1:** Distribution of ESR Intron 1 Pvu II Polymorphism frequencies in migraineurs and controls

	**Genotypes**				**Alleles**	
	**CC**	**CT**	**TT**	**n alleles**	**C**	**T**

**Migraine**	**55 (24%)**	**122 (53%)**	**54 (23%)**	**462**	**232 (50%)**	**230 (50%)**
MA	29 (20%)	84 (58%)	32 (22%)	290	142 (49%)	148 (51%)
MO	22 (30%)	33 (45%)	18 (25%)	146	77 (53%)	69 (47%)
MA & MO	4	5	4	26	13 (50%)	13 (50%)
Male	17 (27%)	31 (48%)	16 (25%)	128	65 (51%)	63 (49%)
Female	38 (23%)	91 (54%)	38 (23%)	334	167 (50%)	167 (50%)
						
**Control**	**46 (23%)**	**97 (48%)**	**59 (29%)**	**404**	**189 (47%)**	**215 (53%)**
Male	12 (19%)	27 (44%)	23 (37%)	124	51 (41%)	73 (59%)
Female	34 (24%)	70 (50%)	36 (26%)	280	138 (49%)	142 (51%)

Statistical analysis of the ESR1 C325G SNP also revealed no significant difference between genotyped migraineurs and the matched control group with regard to genotype frequencies (χ^2 ^= 4.19, *P *= 0.12) and allele frequencies (χ^2 ^= 0.86, *P *= 0.36). Furthermore, no significant difference was seen when the migraine population was subdivided into MA (genotype frequencies χ^2 ^= 5.26, *P *= 0.07, allele frequencies χ^2 ^= 1.67, *P *= 0.20) and MO (genotype frequencies χ^2 ^= 1.15 *P *= 0.56, allele frequencies χ^2 ^= 0.02, *P *= 0.90), and males (genotype frequencies χ^2 ^= 2.54, *P *= 0.28, allele frequencies χ^2 ^= 0.07, *P *= 0.79) and females (genotype frequencies χ^2 ^= 3.05, *P *= 0.22, allele frequencies χ^2 ^= 1.53, *P *= 0.22). Allele frequencies did not deviate from Hardy Weinberg Equilibrium in both case and control groups (at *P *= 0.1, *P *= 0.3) and were similar to frequencies previously reported in an Australian study group [25]. Frequency distribution appears in Table 2.

**Table 2:** Distribution of ESR Exon 4 Codon C325G Polymorphism frequencies in migraineurs and controls

	**Genotypes**				**Alleles**	
	**CC**	**CG**	**GG**	**n alleles**	**C**	**G**

**Migraine**	**133 (58%)**	**90 (39%)**	**8 (3%)**	**462**	**356 (77%)**	**106 (23%)**
MA	77 (55%)	59 (42%)	5 (3%)	282	213 (76%)	69 (24%)
MO	47 (62%)	26 (35%)	2 (3%)	150	120 (80%)	30 (20%)
MA & MO	9	5	1	30	23 (77%)	7 (23%)
Male	39 (61%)	24 (37%)	1 (2%)	128	102 (80%)	26 (20%)
Female	94 (56%)	66 (40%)	7 (4%)	334	254 (76%)	80 (24%)
						
**Control**	**160 (64%)**	**76 (31%)**	**13 (5%)**	**498**	**396 (79%)**	**102 (21%)**
Male	38 (63%)	18 (30%)	4 (7%)	120	94 (78%)	26 (22%)
Female	122 (64%)	58 (31%)	9 (5%)	378	302 (80%)	76 (20%)

### Linkage Disequilibrium analysis

There was no evidence for pairwise linkage disequilibrium between the exon 4 and exon 8 markers and the intron 1 and exon 8 markers. There was however, evidence for linkage disequilibrium between the intron 1 and exon 4 markers (D' = 0.268, p = 0.0001). Table 3 shows D' and *P *values generated by the LD analysis as well as the physical distance between the markers. The distance calculations were performed using information on genomic location of the relevant SNP provided by Ensembl v.34, Oct 2005 [36].

**Table 3:** Linkage disequilibrium D' values (upper right hand side) and distance in bases between markers (lower left hand side).

**Marker**	**PvuII**	**C325G**	**G594A**
**PvuII**	-	D' = 0.268 *P *= 0.0001	D' = 0.016 *P *= 0.52
**C325G**	102188 bases	-	D' = 0.060 *P *= 0.71
**G594A**	256761 bases	154573 bases	-

## Conclusion

The human ESR1 gene is located on chromosome 6q25.1 and contains 8 exons [31]. It is widely expressed in a broad range of tissues including CNS areas such as the hypothalamus, limbic system, hippocampus, cortices of the temporal lobe and the brainstem [37]. Numerous studies have demonstrated the multifunctional role of the ESR, particularly in the CNS. It is understood to play a role in neuroprotection via activation of the MAPK pathway [38], as well as in cognition, mood, and memory [39]. ESR can be activated by neurotransmitters and growth factors, in particular, dopamine [40]. Estrogens can induce Ca^2+ ^mobilization, and activate several kinases including protein kinase C, and phosphatidylinositol-3-OH kinase [41,42]. Estrogen deficiency has been implicated in pathological and degenerative processes in the CNS, while elevated levels have been involved in the development and progression of tumours [43]. In view of the wide variety of mechanisms under control of estrogen and its cognate receptor, particularly in known migraine pathways in the CNS, as well as the well-known role for hormones in migraine, variation in function of the estrogen receptor gene may play a role in neurological conditions, such as migraine. We have previously reported a role for the ESR1 G594A polymorphism in migraine susceptibility. This study investigated two further synonymous polymorphisms in ESR1, the widely studied *Pvu *II T/C polymorphism in intron 1, and the C325G polymorphism at exon 4.

Results showed no association with migraine in the case-control groups for both the intron 1 *Pvu *II marker and the exon 4 C325G marker. There was no evidence for LD between the exon 4 marker and the previously studied exon 8 marker nor between the intron 1 and exon 8 markers. The physical distance between the loci is ~155 kb for exon 4 and 8 markers, and ~257 kb between intron 1 and exon 8 markers. Absence of linkage disequilibrium at the same exon 4 and 8 loci has previously been reported in a different case-control panel by Curran et al (2001) [25]. Our study showed that there was evidence for linkage disequilibrium between the intron 1 and exon 4 markers which are ~102 kb apart. Similar pairwise LD results between these two loci have been previously reported [44].

The fact that alleles of the two SNPs tested in the present study showed no association with migraine and were not in LD with alleles at the exon 8 SNP highlights the need for further studies with a high density of polymorphisms spanning the estrogen receptor to further investigate our previously reported susceptibility locus at exon 8. In particular, such studies should focus on markers that are in LD blocks with the G594A polymorphism. Additionally, we believe further investigation of the exon 8 locus for a potential functional variant is clearly warranted, perhaps utilising allele specific gene expression methods. Also worthy of note is the recent report of an association of the exon 4 C325G polymorphism with migraine in women in a large Spanish cohort [44]. Although our results did not demonstrate this, there was an interesting trend in the female subgroup which we believe warrants further investigation in a larger study group.

## Competing interests

The author(s) declare that they have no competing interests.

## Authors' contributions

NC carried out the majority of the genotyping and data analysis and drafted the manuscript. The data analysis was supervised by RL, who also helped draft the manuscript. SQ oversaw recruitment, diagnosis, and clinical characterization of the subjects used in this study. LG conceived of the study, and participated in its design and coordination and helped to draft the manuscript. All authors read and approved the final manuscript.

## Pre-publication history

The pre-publication history for this paper can be accessed here:


